# Supplementary material for: Coding of chromatic spatial contrast by macaque V1 neurons
Source: eLife. 2022 Feb 11;11:e68133. doi: 10.7554/eLife.68133 (PMC8920507; doi:10.7554/eLife.68133)
Supplement: Transparent reporting form [file elife-68133-transrepform1.docx]

***eLife’s* transparent reporting form**

We encourage authors to provide detailed information *within their submission* to facilitate the interpretation and replication of experiments. Authors can upload supporting documentation to indicate the use of appropriate reporting guidelines for health-related research (see [EQUATOR Network](http://www.equator-network.org/%20)), life science research (see the [BioSharing Information Resource](https://biosharing.org/)), or the [ARRIVE guidelines](http://www.plosbiology.org/article/info:doi/10.1371/journal.pbio.1000412) for reporting work involving animal research. Where applicable, authors should refer to any relevant reporting standards documents in this form.

If you have any questions, please consult our Journal Policies and/or contact us: [editorial@elifesciences.org](mailto:editorial@elifesciences.org).

**Sample-size estimation**

- You should state whether an appropriate sample size was computed when the study was being designed
- You should state the statistical method of sample size computation and any required assumptions
- If no explicit power analysis was used, you should describe how you decided what sample (replicate) size (number) to use

Please outline where this information can be found within the submission (e.g., sections or figure legends), or explain why this information doesn’t apply to your submission:

Two macaque monkeys were used in these experiments. The number two was not based on a power analysis but is the minimum that affords replication. The number of neurons, 98, is the maximum from which we were able to record within the constraints of the study duration.

**For more details please see MATERIALS & METHODS**

**Replicates**

- You should report how often each experiment was performed
- You should include a definition of biological versus technical replication
- The data obtained should be provided and sufficient information should be provided to indicate the number of independent biological and/or technical replicates
- If you encountered any outliers, you should describe how these were handled
- Criteria for exclusion/inclusion of data should be clearly stated
- High-throughput sequence data should be uploaded before submission, with a private link for reviewers provided (these are available from both GEO and ArrayExpress)

Please outline where this information can be found within the submission (e.g., sections or figure legends), or explain why this information doesn’t apply to your submission:

The distinction between biological and technical replication does not easily map onto the methods used in this study.

Inclusion criteria for individual neurons can be found on page 28 (in the "Cell Screening" section).

Comparisons are made across three types of neurons: simple cells, double-opponent cells, and cells that did not satisfy the criteria for inclusion into either of these categories. The number of neurons in each category is provided on page 7: Twenty-six neurons were classified as simple cells, 27 were classified as DO cells, and 47 neurons were neither simple nor DO and were classified conservatively as “OSO” (other spatially opponent).

Page 10: 95/98 neurons had target firing rates that were greater than the 95th percentile value of their respective baseline firing rate distribution.

**Statistical reporting**

- Statistical analysis methods should be described and justified
- Raw data should be presented in figures whenever informative to do so (typically when N per group is less than 10)
- For each experiment, you should identify the statistical tests used, exact values of N, definitions of center, methods of multiple test correction, and dispersion and precision measures (e.g., mean, median, SD, SEM, confidence intervals; and, for the major substantive results, a measure of effect size (e.g., Pearson's r, Cohen's d)
- Report exact p-values wherever possible alongside the summary statistics and 95% confidence intervals. These should be reported for all key questions and not only when the p-value is less than 0.05.

Please outline where this information can be found within the submission (e.g., sections or figure legends), or explain why this information doesn’t apply to your submission:

Page 7: Pearson’s r between the two sides: -0.94 ± 0.11 (mean ± SD) for simple cells, -0.76 ± 0.23 for DO cells, and -0.77 ± 0.24 for OSO cells.

Page 8: NLIs differed across the three cell types (median white noise NLI for simple cells = 0.0009, DO cells = 0.0005, OSO cells = 0.0034; p=0.02, Kruskal-Wallis test).

Page 8: NLIs of simple and DO cells were both lower than those of the OSO cells (p<0.05, Mann-Whitney U tests).

Page 10: Target firing rates did not differ across cell types (p=0.57, Kruskal-Wallis Test).

Page 11: NLIs of simple cells and DO cells were close to zero and did not differ significantly (median isoresponse NLI for DO cells = 0.1007, median isoresponse NLI for simple cells = -0.0097; p=0.14, Mann-Whitney U test).

Page 11: NLIs were greater for the PSP neurons (median isoresponse NLI = 0.2822, p=0.02, Kruskal- Wallis test).

Page 11: (r = 0.30, p = 0.001, Spearman’s correlation between isoresponse NLI and white noise NLI). This correlation was driven primarily by NSNDO cells (r = 0.41, p = 0.004, Spearman’s rank correlation) and not by DO (r = 0.01, p = 0.95, Spearman’s rank correlation) or simple cells (r = -0.19, p = 0.34, Spearman’s rank correlation).

Page 13: Within-subfield NLIs differed across the three cell types (p<0.0001, Kruskal-Wallis test; Figure 5). On average, within-subfield NLIs were higher for the OSO cells than for the simple or DO cells (median within-subfield NLI for simple cells = -0.0016, median within-subfield NLI for DO cells = -0.0003, median cone signal NLI for other cells = 0.0047, p < 0.0001, Kruskal-Wallis Test).

Page 14: normalized S-cone weight derived from the hyperpixel STA was correlated with isoresponse NLI (r = 0.23, p = 0.02, Spearman’s correlation).

(For large datasets, or papers with a very large number of statistical tests, you may upload a single table file with tests, Ns, etc., with reference to sections in the manuscript.)

**Group allocation**

- Indicate how samples were allocated into experimental groups (in the case of clinical studies, please specify allocation to treatment method); if randomization was used, please also state if restricted randomization was applied
- Indicate if masking was used during group allocation, data collection and/or data analysis

Please outline where this information can be found within the submission (e.g., sections or figure legends), or explain why this information doesn’t apply to your submission:

Group assignments for neurons were based on physiological criteria (see Methods: Spike-triggered covariance analysis). Randomization was not used.

**Additional data files (“source data”)**

- We encourage you to upload relevant additional data files, such as numerical data that are represented as a graph in a figure, or as a summary table
- Where provided, these should be in the most useful format, and they can be uploaded as “Source data” files linked to a main figure or table
- Include model definition files including the full list of parameters used
- Include code used for data analysis (e.g., R, MatLab)
- Avoid stating that data files are “available upon request”

Please indicate the figures or tables for which source data files have been provided:

Data are available at https://github.com/horwitzlab/Chromatic_spatial_contrast
